# Enamel Formation Genes Influence Enamel Microhardness Before and After Cariogenic Challenge

**DOI:** 10.1371/journal.pone.0045022

**Published:** 2012-09-24

**Authors:** Takehiko Shimizu, Bao Ho, Kathleen Deeley, Jessica Briseño-Ruiz, Italo M. Faraco, Brett I. Schupack, João A. Brancher, Giovana D. Pecharki, Erika C. Küchler, Patricia N. Tannure, Andrea Lips, Thays C. S. Vieira, Asli Patir, Mine Yildirim, Fernando A. Poletta, Juan C. Mereb, Judith M. Resick, Carla A. Brandon, Iêda M. Orioli, Eduardo E. Castilla, Mary L. Marazita, Figen Seymen, Marcelo C. Costa, José M. Granjeiro, Paula C. Trevilatto, Alexandre R. Vieira

**Affiliations:** 1 Department of Pediatric Dentistry, Nihon University of Dentistry at Matsudo, Matsudo, Chiba, Japan; 2 Department of Oral Biology, University of Pittsburgh, Pittsburgh, Pennsylvania, United States of America; 3 Latin American Collaborative Study of Congenital Malformations (ECLAMC), Center for Medical Education and Clinical Research (CEMIC), Buenos Aires, Argentina; 4 Center for Health and Biological Sciences, Pontifical Catholic University of Paraná (PUCPR), Curitiba, Brazil; 5 Clinical Research Unit, Biology Institute, Fluminense Federal University, Niterói, Rio de Janeiro, Brazil; 6 Discipline of Pediatric Dentistry, Veiga de Almeida University, Rio de Janeiro, Brazil; 7 Department of Pediatric Dentistry and Orthodontics, Federal University of Rio de Janeiro, Rio de Janeiro, Brazil; 8 Department of Pedodontics, Istanbul Medipol University, Istanbul, Turkey; 9 Department of Pedodontics, Faculty of Dentistry, Istanbul University, Istanbul, Turkey; 10 Latin American Collaborative Study of Congenital Malformations (ECLAMC), Hospital de Area El Bolsón, Río Negro, Argentina; 11 Latin American Collaborative Study of Congenital Malformations (ECLAMC), National Institute of Population Medical Genetics (INAGEMP-CNPq), Department of Genetics, Institute of Biology, Center of Health Sciences, Federal University of Rio de Janeiro, Rio de Janeiro, Brazil; 12 Latin American Collaborative Study of Congenital Malformations (ECLAMC), National Institute of Population Medical Genetics (INAGEMP-CNPq), Department of Genetics, Oswaldo Cruz Foundation, Rio de Janeiro, Brazil; 13 Center for Craniofacial and Dental Genetics, Department of Human Genetics, and Clinical and Translational Science, University of Pittsburgh, Pittsburgh, Pennsylvania, United States of America; 14 Center for Craniofacial and Dental Genetics, Department of Pediatric Dentistry, School of Dental Medicine, and Clinical and Translational Science, University of Pittsburgh, Pittsburgh, Pennsylvania, United States of America; University of Southern California, United States of America

## Abstract

There is evidence for a genetic component in caries susceptibility, and studies in humans have suggested that variation in enamel formation genes may contribute to caries. For the present study, we used DNA samples collected from 1,831 individuals from various population data sets. Single nucleotide polymorphism markers were genotyped in selected genes (*ameloblastin*, *amelogenin*, *enamelin*, *tuftelin*, and *tuftelin interacting protein 11*) that influence enamel formation. Allele and genotype frequencies were compared between groups with distinct caries experience. Associations with caries experience can be detected but they are not necessarily replicated in all population groups and the most expressive results was for a marker in *AMELX* (p = 0.0007). To help interpret these results, we evaluated if enamel microhardness changes under simulated cariogenic challenges are associated with genetic variations in these same genes. After creating an artificial caries lesion, associations could be seen between genetic variation in *TUFT1* (p = 0.006) and *TUIP11* (p = 0.0006) with enamel microhardness. Our results suggest that the influence of genetic variation of enamel formation genes may influence the dynamic interactions between the enamel surface and the oral cavity.

## Introduction

Caries continues to be a great burden to individuals, particularly children, families, and society. Studies evaluating genetic variation and its implications to caries have been proposed with the hopes that new discoveries can lead to innovative approaches to prevent the condition [1].

Genes responsible for enamel formation have been proposed as potentially involved in caries susceptibility, and positive associations between genetic variation in *amelogenin*, *tuftelin*, and *enamelin* and higher caries experience have been reported [2]–[4]. These results are promising because now one can propose that variation in the enamel surface could predispose individuals to development of carious lesions. The identification of individuals with “caries-predisposing enamel” not only would allow for personalizing preventive strategies, but also provide support for the popular belief of a correlation between “weak teeth” and having many “cavities.”

It is well documented that the first studies of complex traits suggest a stronger genetic effect than is found by subsequent studies. Both bias and genuine population diversity might explain why early studies tend to overestimate the disease predisposition conferred by candidate gene polymorphisms [5]. Our hypothesis is that variation in genes involved in enamel formation contributes to increased caries susceptibility in humans. Therefore, we investigated genetic markers in genes involved in enamel formation that were previously studied [2]–[4] to provide independent replication of the original results, which suggested that variation in *amelogenin*, *tuftelin*, and *enamelin* contribute to higher caries experience in humans. Also, we tested if enamel microhardness varies depending on genetic variation and cariogenic challenge to unveil a possible mechanism for higher caries susceptibility related to enamel structure.

## Results

### Association Results in the Filipino Families

All genotypes were in Hardy-Weinberg equilibrium. Markers studied that were in the same gene were in weak linkage disequilibrium (data not shown). Statistically significant association between the intronic marker rs946252 of *AMELX* and caries experience (p = 0.0007; T allele over transmitted) was detected by transmission disequilibrium tests (Table 1).

**Table 1:** Family-based association test results for caries experience in Filipino familes.

marker	chromosome	gene	region	Allele (frequency)	Informative families	P-value
rs946252	X	*AMELX*	Intron 2	C/T (0.63/0.37)	25	**0.0007**
rs4970957	1	*TUFT1*	Intron 1	A/G (0.55/0.45)	36	0.67
rs4694075	4	*AMBN*	Intron 5	C/T (0.32/0.68)	35	0.20
rs12640848	4	*ENAM*	Intron 8	A/G (0.80/0.20)	24	0.38
rs5997096	22	*TFIP11*	Intron 7	C/T (0.43/0.57)	37	0.46

Statistically significant p-values (p<0.05) are presented in bold font.

When analysis was performed based on allele frequency differences, statistical significant association was detected for rs946252 in *AMELX* (Table 2). The frequency of the T allele of rs946252 in the high caries experience group was significantly higher than that in the low caries experience group, (C : T = 357∶ 241 in high caries group, C : T = 242∶114 in low caries group, p = 0.01). The *AMBN* rs4694075 was also associated with caries experience. The frequency of the C allele of this marker in high caries experience individuals was significantly higher than that in the low caries experience group, (C : T = 210∶ 388 in high caries experience group, C : T = 105∶285 in low caries experience group, p = 0.007).

**Table 2:** Association results for caries experience in Filipinos and in the replication sample sets.

	PHILIPPINES		TURKEY		ARGENTINA		BRAZIL (Curitiba)		BRAZIL (Rio de Janeiro)	
MarkerandGene	genotypep-value	allelep-value	genotypep-value	allelep-value	genotypep-value	allelep-value	genotypep-value	allelep-value	genotypep-value	allelep-value
rs946252 *AMELX*	0.11	**0.01**	0.10	**0.04**	0.26	0.2	0.43	0.22	0.69	0.31
rs4970957 *TUFT1*	0.32	0.53	0.75	0.5	0.07	**0.03**	0.54	0.8	0.17	**0.04**
rs4694075 *AMBN*	0.14	**0.007**	0.16	0.29	0.6	0.29	0.33	0.22	0.43	0.94
rs12640848 *ENAM*	0.49	0.52	0.52	0.73	0.87	0.76	0.19	**0.04**	0.58	0.29
rs5997096 *TFIP11*	0.93	0.76	0.77	0.61	0.96	0.78	0.86	0.61	0.49	0.24

P-values <0.05 are presented in bold font.

### Replication Studies

Replication studies showed trends of association with all genes studied with the exception of *TFIP11.* In Turkish children, statistically significant association with caries experience was detected for rs946252 in *AMELX* (Table 2). The T allele of this marker was significantly more frequent in the caries group in comparison to the caries free group, (C : T = 47∶ 133 in the caries group, C : T = 62∶ 110 in the caries free group, p = 0.04). This result replicates the findings in the Filipino families.

In the Argentina cohort, statistically significant association between rs4970957 in *TUFT1* and caries experience was detected (A : G = 85∶ 33 in the high caries experience group, A : G = 91∶ 61 in the low caries experience group, p = 0.03). This result replicates previous findings in Iowa, US [2] and Guatemala [3] (Table 3).

**Table 3:** Previously reported associations between enamel formation genes and caries susceptibility.

Genes Studied	Stayton et al. [2005]	Deeley et al. [2008]	Patir et al. [2008]
*Ameloblastin*	−	−	+
*Amelogenin*	−	+	+
*Enamelin*	−	−	+*
*Tuftelin*	+*	+#	+
*Tuftelin interacting protein 11*	−	−	−

* In the presence of *Streptococcus mutans*.

# Only is less severely affected cases.

In the Brazilian cohort from Curitiba, statistically significant association between rs12640848 in *ENAM* and caries experience was detected (A : G = 101∶ 157 in high-caries group, A : G = 266∶ 554 in low-caries group, P = 0.04). This result replicates previous findings in Turkey [4] (Table 3).

In the Brazilian cohort from Rio de Janeiro, statistically significant association between rs4970957 in *TUFT1* and caries experience was detected (A : G = 263∶ 69 in the high caries experience group, A : G = 463∶ 85 in the low caries experience group, p = 0.04). This result replicates findings in Iowa, US [2], Guatemala [3], and Turkey [4] (Table 3).

### Mutation Analysis

We identified three silent mutations that do not result in a change to the amino acid sequence of a protein, one in exon 6 of *AMELX* (c.361C>T, rs2106416) and two at the exon 10 of *AMBN* (c.657A>G, rs35266919 and c.678C>T, rs113360877). We also identified one non-synonymous mutation in exon 1 of *TUFT1* (c.53A>G in TUFT1, rs3828054). These sequence variants are all included in the Ensembl cDNA Report (http://www.ensembl.org/index.html).

No significant differences in the frequency of alleles were detected between caries affected and caries free groups [c.262 C>T in *AMELX* exon 6 (C : T = 76∶ 10 in the caries affected group, C : T = 42∶ 8 in the caries free group, p = 0.47), c.53A>G (Glu18Arg) in *TUFT1* exon 1 (A : G = 75∶ 7 in the caries affected group, A : G = 44∶ 2 in the caries free group, p = 0.37), c.657A>G in *AMBN* exon 10 (A : G = 74∶ 12 in the caries affected group, A : G = 45∶ 7 in the caries free group, p = 0.94), and c.678C>T in *AMBN* exon 10 (C : T = 83∶ 3 in the caries affected group, C : T = 51∶ 1 in the caries free group, p = 0.58)]. No mutations were detected in the 5′-upstream region (2232 base pairs) of *AMELX* or any other coding regions of the five genes studied.

### Enamel Microhardness Studies

As expected, enamel microhardness decreased after creation of artificial caries lesions and then increased to levels similar to baseline after the one-time fluoride treatment and the pH-cycling protocol for 14 days (Table 4). These trends were present in all types of samples. Correlations were statistically significant after the creation of artificial caries in enamel blocks from distal (correlation coefficient = 0.44) and occlusal surfaces (correlation coefficient = 0.47). Statistically significant negative correlation between enamel microhardness after fluoride treatment in occlusal surfaces (correlation coefficient = −0.73) was detected. Statistically significant negative correlation between enamel microhardness after pH-cycling treatment and distal surfaces (correlation coefficient = −0.42) was also detected.

**Table 4:** Single marker association results for enamel microhardness.

BASELINE													
	Mesial			Distal		Buccal*					Occlusal		
Marker and Gene	Genotypep-value	Allelep-value		Genotypep-value	Allelep-value	Genotypep-value			Allelep-value		Genotypep-value		Allelep-value
*AMELX*													
rs17878486	0.13	0.36		0.52	0.22	0.14			**0.03**		0.30		0.06
rs946252	0.58	0.28		0.20	0.82	0.57			0.28		0.39		0.17
*TUFT1*													
rs3790506	0.11	**0.03**		0.42	0.81	0.09			**0.02**		0.07		0.13
rs2337360	0.31	0.14		0.43	0.23	0.21			0.24		0.26		0.08
rs4970957	0.52	0.96		0.06	0.10	1			1		0.22		0.27
*AMBN*													
rs34538475	0.43	0.23		**0.02**	**0.04**	0.22			1		0.39		1
rs4694075	0.11	0.06		0.56	0.43	0.26			0.11		0.28		1
*ENAM*													
rs3796704	0.76	0.34		0.13	0.74	0.57			0.35		0.39		0.13
rs12640848	0.26	0.44		0.87	0.65	0.54			0.66		0.81		0.67
*TFIP11*													
rs134136	0.61	0.69		0.37	0.79	0.40			0.09		0.22		0.38
rs5997096	0.28	0.46		0.34	0.79	0.22			0.22		1		1
**ARTIFICIAL CARIES/BASELINE**													
	**Mesial**			**Distal**		**Buccal**					**Occlusal**		
**Marker and Gene**	**Genotype** **p-value**	**Allele** **p-value**		**Genotype** **p-value**	**Allele** **p-value**	**Genotype** **p-value**			**Allele** **p-value**		**Genotype** **p-value**		**Allele** **p-value**
*AMELX*													
rs17878486	0.31	0.07		0.57	0.28	0.57			0.28		0.36		0.53
rs946252	0.36	0.54		0.80	0.58	0.21			1		0.39		0.17
*TUFT1*													
rs3790506	0.34	0.19		0.22	0.06	0.09			**0.02**		1		1
rs2337360	0.56	0.07		**0.01**	**0.006**	0.84			0.68		0.54		0.38
rs4970957	1	1		0.57	0.62	0.23			0.28		0.22		0.27
*AMBN*													
rs34538475	0.56	0.69		1	1	0.56			0.40		0.39		0.17
rs4694075	0.78	0.46		0.49	0.25	0.26			0.11		0.78		0.38
*ENAM*													
rs3796704	0.76	0.70		0.51	0.62	0.22			0.68		0.50		0.53
rs12640848	0.26	0.44		0.09	**0.02**	0.54			0.66		0.81		0.67
*TFIP11*													
rs134136	0.61	0.69		0.40	0.22	**0.01**			**0.0006**		**0.05**		**0.009**
rs5997096	0.55	0.46		0.22	0.27	0.57			0.68		0.34		0.20
**FLUORIDE/ARTIFICIAL CARIES**													
	**Mesial**			**Distal**		**Buccal**					**Occlusal**		
**Marker and Gene**	**Genotype** **p-value**	**Allele p-value**		**Genotype** **p-value**	**Allele** **p-value**	**Genotype** **p-value**			**Allele** **p-value**		**Genotype** **p-value**		**Allele** **p-value**
*AMELX*													
rs17878486	1	1		0.57	0.28	0.21			1		0.30		0.06
rs946252	0.93	0.54		0.17	1	0.57			0.28		0.51		0.61
*TUFT1*													
rs3790506	0.58	0.49		0.27	1	0.19			0.66		0.07		0.13
rs2337360	0.56	1		0.84	0.69	0.30			0.24		0.13		**0.03**
rs4970957	1	1		0.57	0.62	1			1		1		1
*AMBN*													
rs34538475	0.56	0.69		1	1	0.40			1		0.54		0.38
rs4694075	0.78	0.46		0.49	0.25	0.78			0.43		0.16		0.08
*ENAM*													
rs3796704	0.76	0.70		0.51	0.62	0.22			0.06		0.51		0.61
rs12640848	1	1		0.24	0.12	0.42			0.19		0.81		0.67
*TFIP11*													
rs134136	0.13	0.23		**0.02**	0.22	0.40			0.22		0.22		0.38
rs5997096	0.26	0.14		0.31	0.22	0.57			0.68		1		1
**pH-CYCLING/FLUORIDE**													
	**Mesial**		**Distal**				**Buccal**			**Occlusal**			
**Marker and Gene**	**Genotype p-value**	**Allele p-value**	**Genotype p-value**		**Allele p-value**		**Genotype p-value**	**Allele p-value**		**Genotype p-value**		**Allele p-value**	
*AMELX*													
rs17878486	1	1	0.57		0.28		0.14	**0.03**		0.30		0.06	
rs946252	0.07	**0.03**	0.30		0.35		0.14	**0.03**		0.51		0.65	
*TUFT1*													
rs3790506	0.42	0.66	0.42		0.19		0.19	0.66		0.55		0.61	
rs2337360	1	1	0.30		0.24		0.84	0.69		0.30		0.20	
rs4970957	0.24	0.28	1		1		1	1		**0.01**		**0.02**	
*AMBN*													
rs34538475	0.56	0.69	0.28		0.28		0.40	1		0.06		0.38	
rs4694075	0.14	0.14	0.81		0.70		0.78	0.43		0.78		0.38	
*ENAM*													
rs3796704	0.59	0.26	0.42		0.13		0.34	1		0.39		0.13	
rs12640848	0.28	0.12	0.54		0.24		0.54	0.66		0.16		**0.01**	
*TFIP11*													
rs134136	0.13	0.23	0.42		1		0.08	**0.04**		**0.05**		**0.009**	
rs5997096	1	1	0.40		0.22		0.57	0.68		0.26		0.40	

p-values <0.05 are presented in bold font.

* Samples from buccal and lingual surfafes were analyzed together.

Artificial Caries/Baseline; The ratio of change for microhardness after creation of artificial caries.

Fluoride/Artificial Caries; The ratio of change for microhardness after fluoride treatment.

pH-cycling/Fluoride; The ratio of change for microhardness after pH-cycling treatment.

Lower baseline microhardness was significantly associated with rs17878486 in *AMELX* (p = 0.03 for buccal surface), rs3790506 in *TUFT1* (p = 0.03 for mesial and p = 0.02 for buccal + lingual surfaces), and rs34538475 in *AMBN* (p = 0.04 for distal surface). After artificial caries creation, lower microhardness was significantly associated with rs3790506 in *TUFT1* (p = 0.02 for buccal + lingual surfaces), rs2337360 in *TUFT1* (p = 0.006 for distal surface), rs1260848 in *ENAM* (p = 0.02 for distal surface) and rs134136 in *TFIP11* (p = 0.0006 for buccal + lingual and p = 0.009 for occlusal surfaces). After fluoride treatment, microhardness was significantly associated with rs2337360 in *TUFT1* (p = 0.03 for occlusal surface). The ratio of change of microhardness after pH-cycling treatment was significantly associated with rs17878486 in *AMELX* (p = 0.03 for buccal + lingual surface), rs946252 in *AMELX* (p = 0.03 for mesial and p = 0.03 for buccal + lingual surfaces), rs4970957 in *TUFT1* (p = 0.02 for occlusal surface), rs12640848 in *ENAM* (p = 0.01 for occlusal surface), and rs134136 in *TFIP11* (p = 0.04 for buccal + lingual and p = 0.009 for occlusal surfaces).

According to our correlation analysis (summarized on Table 5), samples with lower enamel microhardness had greater rates of demineralization in the distal and buccal surfaces (correlation coefficient = 0.43 and 0.79, respectively). When fluoride was applied, samples with higher values of enamel microhardness had greater increases in enamel microhardness in the mesial and buccal + lingual surfaces (greater rates of fluoride uptake, correlation coefficient = 0.34 and 0.66, respectively). After the demineralization-remineralization challenge, positive correlation could be seen for samples from the mesial and occlusal surfaces (correlation coefficient = 0.45 and 0.25, respectively). Enamel microhardness values did not correlate with caries experience of the individuals that provided samples (data not shown).

**Table 5:** Correlations of enamel microhardness values in the several experimental points.

Surface	Artificial Caries/Baseline	Fluoride/Artificial Caries	pH-cycling/Fluoride
Mesial	0.19	0.34	**0.45**
Distal	**0.43**	−0.17	0.04
Buccal (+lingual)	**0.79**	**0.66**	−0.09
Occlusal	−0.09	0.06	0.25

Statistically significant correlations are presented in bold font.

## Discussion

Our results continue to support a role for genes involved in enamel formation in caries, but the individual contributions of these genes is likely to be small. It is also likely that the phenotype definition we used (caries experience based on DMFT scores) is not sensitive enough to precisely detect these effects. This index, which was designed to be applied at 12 years of age, do not offer much insight into the disease process, particularly if taken in adults. One alternative for the DMFT/DMFS scores is the utilization of the International Caries Assessment and Detection System (ICDAS) clinical visual criteria (http://www.icdas.org/). In this system, carious lesions are defined on a scale from sound (healthy surface) to demineralization involving the inner third of dentine, possibly with pulp involvement. This more detailed assessment may provide the opportunity to analyze the data based on severity and extension of lesions, although it is still limited in the sense of translating how lesions are progressing. The best approach may be prospectively or retrospectively obtaining caries experience data (DMFT/dmft, DMFS/dmfs, or ICDAS) at several time points from the same individuals. These data may allow us to infer how disease is progressing, despite the fact that likely subjects will have received the standard of dental care overtime. More discreet groups with more aggressive disease progression may be identified and opportunities to identify host factors influencing disease may be created.

Another limitation of our work is the lack of specific data on bacterial colonization. Selected study groups come from areas where they have similar access to oral care and are under a similar set of cultural norms related to dietary and oral hygiene habits, particular the families from the Philippines [6]. We have previoulsy shown that the presence of *Streptococcus mutans* colonization appears to not interact with genetic variation in genes related to enamel formation [3], a result that disagrees with the initial suggestions that variation in *TUFT1* in the presence of Streptoccocus mutans increases caries experience [2]. When we reanalyzed our data on the presence of *Streptococcus mutans*, we found that it correlates very well with caries experience in the primary dentition, but not in the permanent dentition [7]. Since most of the studied samples here are from adults, we did not include these data in the analysis.

The data sets we studied are not identical and demographic, regional, and cultural differences are likely playing a role in our results. Caries experience in the Brazilian cohorts is much lower than in the other populations. The Turkish data is from primary dentition and a matched caries free control group was used. Data from Argentina and the Philippines are mostly from individuals older than 12 years of age. The only consistent result in our study and the previously published data [2]–[4] is the lack of association between caries experience and variation in *TFIP11*. Our data suggest that associations can be detected between caries experience and *AMELX*, *TUFT1*, *AMBN*, and *ENAM*. These results are not consistent however, and at least are in part due to the differences in the studied samples as mentioned above and other unknown confounders. Genetic changes leading to abnormal protein function or decreased amounts of protein could lead to some degree of disorganization of the enamel prisms that increases the individual’s susceptibility to caries. However, testing the hypothesis that an intronic single nucleotide polymorphism (SNP) changes protein expression is not a simple task. Testing amino acid-altering coding SNPs for their effect on gene transcription often requires elaborate expression constructs and analysis using an *in vitro* system, however this approach does not allow testing the functionality of SNPs in intronic regions, unknown regulatory elements, or intergenic regions [8]. Furthermore, our underlying hypothesis is that enamel development is affected to the point it increases caries risk, and it is difficult to conceive an experiment that allow to directly test if enamel that developed under variable levels of gene expression will be more susceptible to demineralization, without generating several lines of hypomorphic mice.

To help interpret these results, we decided to design a series of functional assays to evaluate the response of enamel samples with known genotypes of the genes involved in enamel formation to simulated cariogenic challenges.

Despite the limitation of having samples from several different types of teeth (first, second, and third molars, premolars, canines, and incisors), the results of these experiments suggest that that may be some truth on the popular belief that some individuals may have “weaker” teeth, and hence are more prone to caries development. We also found correlations suggesting that depending on the tooth surface, softer enamel may demineralize easier and harder enamel may be better in acquiring fluoride after exposure to fluoridated toothpaste. Suggestive associations were found between enamel microhardness and *TUFT1*, *AMELX*, and *AMBN*, particularly in case of smooth surfaces (buccal, lingual, and proximal). These results are in part supported by independent analyses suggesting that distinct genetic factors may exert different effects on caries development in smooth versus occlusal surfaces [9]. After creating an artificial caries lesion, associations could be seen between genetic variation in *TUFT1* (buccal + lingual surfaces) and *TUIP11* (buccal + lingual, distal, and occlusal surfaces) and enamel microhardness. This result is intriguing since it suggests that genetic variation may be linked to enamel surfaces that more easily demineralize. In this regard, it is also remarkable that genetic variation in *TUIP11*, which we and others do not find associated with high caries experience, is associated with subclinical demineralization. These variants were also associated with fluoride uptake in the pH-cycling protocol. *TUIP11* appears to participate in multiple cellular activities as diverse as pre-mRNA splicing, cell cycle activity and tumorigenesis [10]. We can speculate that genetic variation in *TUIP11* may influence the ability of the enamel surface’s ability of uptaking fluoride, even in very low concentrations. This effect may decrease individual susceptibility to demineralization at subclinical levels. These protective effects, however, can be easily overcome, which makes the detection of any effects of *TUIP11* in caries unlikely if one is defining the phenotype based solely on caries experience. Genetic variation in *TUFT1* (occlusal surface) and *AMBN* (distal surface) may allow for more efficient fluoride uptake when higher concentrations of fluoride are present (*i.e*., the use of fluoridated toothpaste during tooth brushing). Since caries experience of the individuals whose samples were tested for microhardness did not correlate with the results, phenotypes not assessed and studied here such as enamel thickness, crystallite size and morphology, enamel composition, acid liability, and others could provide a better understanding of the role of genetic variation in enamel formation genes in caries susceptibility.

Another observation in our study is that enamel microhardness varies from individual to individual, sometimes substantially. Traditional protocols avoid these variations by eliminating samples that are outside a specific range {*i.e.*, [11] limited their study to specimens with knoop microhardness values between 350 and 380}. Although this methodological approach reduces inter-specimens variation, it also eliminates the chance of interpreting the results in light of individual variation. Our results suggest that the influence of genetic variation of enamel formation genes may influence the dynamic interactions between the enamel surface and the oral cavity. Components not studied here include biofilm formation (both adhesion to the enamel and maturation), and the influence of salivary components. Despite these limitations, the determination of the presence of specific genetic variants in patients holds the promise for allowing customized treatments that may better impact individual risks for caries.

## Materials and Methods

### Subjects and Definition of Caries Experience Level

The study populations consisted of 1,831 individuals from five populations including Filipino, Turkish, Argentinean, and two Brazilian cohorts. Caries experience was defined as described previously [6] and saliva sample collection and DNA extractions were described previously [2], [3], [6], [12], [13]. The Filipino population consisted of 477 subjects (224 females and 253 males) from 72 pedigrees living in the Cebu island, in an attempt to reduce the influence of environmental factors. This study protocol was approved by the University of Pittsburgh and H.O.P.E. Foundation International Institutional Review Boards, and written informed consent was obtained from all participants. The mean age of the population was 25.8 years-old and ranged from 1 to 82. The mean DMFT score was 9.7 and ranged from 0 to 32. In our study, the population was classified as either ‘low caries group’ or ‘high caries group,’ based on DMFT scores and age (Table 6). The criteria used here for classification of caries experience took age into consideration, since it was expected that caries experience will increase in the general population with age [14]. DMFT cut-offs were modified from the World Health Organization Oral Health Report of 2003 [15]. We have previously tested variations of these cut-off definitions, and the results suggest that these variations do not affect the findings (data not shown).

**Table 6:** Definitions of caries experience based on age and DMFT (Decayed, Missing due to caries, Filled Teeth) scores used in the Filipino families.

Caries Experience Level	DMFT^a^	No. of individualsMean ± SD^b^	DMFT Mean ± SD^b^	Age
Children [up to 12 yrs of age]		115	7.5±5.4	8.4±2.8
Low caries experience:	0–2	26	1.0±0.9	7.0±3.5
High caries experience:	3 or higher	89	9.4±4.7	8.8±2.4
Teenagers [from 13 to 19 yrs of age]		104	8.0±5.9	15.8±1.9
Low caries experience:	0–5	44	3.0±1.4	15.5±1.8
High caries experience:	6 or higher	60	11.5±5.2	16.0±1.9
Adults [20 yrs of age and older]		258	11.4±8.0	37.7±13.0
Low caries experience:	0–8	109	4.4±2.4	33.0±10.8
High caries experience:	9 or higher	149	16.5±6.7	41.1±13.5
Total		477	9.7±7.3	25.8±16.3

a DMFT cut-offs were modified from the World Health Organization (World Health Organization, 2003),

b Standard deviation.

Details regarding the demographics and caries experience of the five study groups are presented in Table 7. With the exception of Brazil, drinking water in the regions where samples originated is not artificially fluoridated.

**Table 7:** Demographics and caries experience of the replication study populations.

	Philippines	Turkey	Argentina	Brazil (Curitiba )	Brazil (Rio de Janeiro)
Sample size (mean DMFT^a^ ± SD^b^)	477 (9.7±7.3)	172 (3.8±4.0)	143 (7.1±7.8)	539 (1.4±1.9)	500 (2.4±3.0)
High caries group^c^ (mean DMFT ± SD)	298 (13.3±6.7)	92 (7.2±2.3)	66 (13.0±7.9)	117 (4.4±1.5)	171 (5.8±2.6)
Low caries group^c^ (mean DMFT ± SD)	179 (3.6±2.4)	80 (0)	77 (2.0±2.3)	410 (0.6±0.9)	329 (0.6±0.9)
Females	224	93	83	261	236
Males	253	79	60	278	264
Age (mean ± SD)	25.8±16.3	5.4±0.8	21.7±15.6	12.0±0.5	9.1±3.1
The number of pedigrees	72	unrelated	unrelated	unrelated	unrelated

a Decayed, Missing due to caries, Filled Teeth.

b Standard deviation.

c High and low caries experience was defined based on criteria 1 on Table 1.

The study sample from Istanbul, Turkey, consisted of 172 unrelated children (93 females and 79 males) from 3 to 6 years of age. Ninety children had a dmft score of four or more and 82 children were caries-free [4]. Both Istanbul University and University of Pittsburgh Institutional Review Boards approved the study of these samples and written informed consent was obtained from the parents of all participants.

From Argentina, 143 DNA samples from unrelated individuals (83 females and 60 males) living in twelve Patagonia sites were studied (San Carlos de Bariloche, El Bolsón, Esquel, El Maitén, Maquinchao, Ingeniero Jacobacci, Rio Colorado, Choele Choel, Valcheta, Sierra Grande, Santo Antonio Oeste, and General Roca), as part of our ongoing studies on genetic susceptibility to isolated cleft lip and palate. No individuals born with clefts were included in the analysis. The mean age was 21.7 years (between infants under 1 and 72 years with median of 18 years) and both the Centro de Educación Médica e Investigaciones Clínicas “Norberto Quirno” (CEMIC) and University of Pittsburgh Institutional Review Boards approved the study of these samples and written informed consent was obtained from all participants (parents provided consent for the participation of individuals 17 years of age and under).

From Brazil, two sample data sets were available for this study. The first consisted of 539 unrelated children living in Curitiba (261 females and 278 males) with the mean age of 12 years (between 10 and 14 years). These samples were used with the approval of both the Pontifical Catholic University of Paraná and the University of Pittsburgh Institutional Review Boards. Age appropriate assent documents were used for all children and informed, written consent was obtained from the parents. The second group of DNA samples were from 500 unrelated subjects living in Rio de Janeiro (236 females and 264 males) with mean age of 9 years (between 2 and 21 years). These samples were used with the approval of both the Human Ethics Committee of the Health Department of the city of Rio de Janeiro and the University of Pittsburgh Institutional Review Boards. Age appropriate assent documents were used for children between 7 and 14 years and informed; written consent was obtained from the child, as well as from the parents.

Caries experience data (DMFT/dmft) was collected according to established protocols. In the case of data from Turkey, one of the authors (A.P.) carried out the clinical examination after being calibrated by an experienced specialist (F.S.). The intra-examiner agreement was assessed by a second clinical exam in 10% of the sample after 2 weeks, with a κ of 1.0. In the case of Argentina, data was collected by one single experienced specialist examiner (A.R.V.). Subjects in these projects were seen over a period of no longer than three to five days and intra-examiner agreement data could not be generated. Samples from Rio de Janeiro, Brazil, were collected by two examiners (E.C.K. and P.N.T.) and calibrated by an experienced specialist (M.C.C.). The intraexaminer agreement was assessed by a second clinical exam in 20 children after 2 weeks, with a κ of 0.99. Cohen’s kappa values for agreement between examiners were 0.91. Finally, caries experience data from Curitiba, Brazil, were collected by two examiners (J.A.B. and G.D.P.) and calibrated by an experienced specialist (P.C.T.). Inter- and intra-examiner reproducibility was taken on 10% of the sample and κ were 0.93 for inter- and 0.99 for intra-examiner reliability.

### Single Nucleotide Polymorphism (SNP) Genotyping

Five single nucleotide polymorphisms (SNPs) were selected in intronic regions of enamel development genes including rs946252 in *AMELX*, rs4970957 in *TUFT1*, rs4694075 in *AMBN*, rs12640848 in *ENAM* and rs5997096 in *TFIP11*. These SNPs were chosen based on known allele frequencies in Asian populations and to be independent of the SNPs previously studied [2]–[4]. Polymerase chain reactions with TaqMan SNP Genotyping Assays (Applied Biosystems) held in total 5 uL/reaction with 3.0 ng DNA were used for genotyping all selected markers in a Tetrad PTC225 thermocycler (MJ Research, Waltham, MA, USA). Genotype detection and analysis were performed on the ABI 7900HT with ABI SDS software (Applied Biosystems).

Association between caries experience and the SNPs was tested with the transmission disequilibrium test (TDT) within the programs Family-Based Association Test (FBAT) [16]. The pair-wise linkage disequilibrium (LD) between markers was estimated with D’ using the Graphical Overview of Linkage Disequilibrium (GOLD) software [17]. Also, the differences in genotype and allele frequencies between high and low caries groups (caries free and caries affected groups in Turkish children) were tested by chi-square tests. P-values were calculated using the Fisher’s exact test.

### Mutation Analysis

We sequenced all exons, exon-intron boundaries, and untranslated regions of *AMELX*, *AMBN*, and *TUFT1* in the Turkish samples since our previous data suggested these genes were associated with high caries experience in this data set [4].

Individuals with at least one copy of associated alleles were selected. DNA samples from 70 individuals (45 with high caries experience and 25 with low caries experience) were used for sequencing *AMELX*, 69 individuals (43 with high caries experience and 26 with low caries experience) were used for sequencing the *AMBN*, and 64 individuals (41 with high caries experience and 23 individuals with low caries experience) were used for sequencing *TUFT1*.

Primer sets and PCR conditions previously described for *AMELX* [18] and *AMBN* [19] were used. We designed primer sets for amplification of 5′-upstream region of *AMELX*: Forward; GCTAGCCAGACAGTTAAGAG, Reverse; TGGCCAAGAAAGACCTTTGG (product size 1207 base pairs) and Forward; AGCTGTGTAAGTGGGGCCTA, Reverse; CTCTTAACTGTCTGGCTAGC (product size 1326 base pairs). PCR products were directly sequenced using ABI PRISM BigDye™ Terminator Cycle Sequencing Ready Reaction Kit (Applied Biosystems) and ABI 3730xl DNA Analyzer (Applied Biosystems). Sequences obtained were verified against the sequences in the Ensembl (Ensembl Transcript ID, *AMELX*, ENST00000380712; *TUFT1*, ENST00000368849; *AMBN*, ENST00000322937) and two unrelated CEPH (Foundation Jean Dausset-Centre d'Etude du Polymorphisme Humain) DNA controls.

### Enamel Microhardness Analysis

Enamel samples from 48 extracted permanent teeth (34 molars, seven premolars, five mandibular incisors, and two maxillary canines) from 28 subjects participating in the University of Pittsburgh School of Dental Medicine Dental Registry and DNA Repository were used to test the correlation between enamel microhardness at baseline and after creating an artificial caries lesion and genetic variation in the enamel development gene markers described above. The Dental Registry and DNA Repository of the School of Dental Medicine, University of Pittsburgh was approved by the University of Pittsburgh Institutional Review Board (IRB #0606091). Written informed consent was obtained from all participants. Teeth were extracted due to irreversible destruction due to caries or advanced periodontal disease and not all surfaces had enamel strcture available. These subjects had a mean age of 42.3 years, 15 were females and 13 males. In addition, 14 were African Americans, 13 were white, and one was of Asian descent. Their DMFT mean was 13.5 and DMFS mean 48.8. Tooth samples were stored in 2% formaldehyde at room temperature until initial laboratory manipulation. At the start of sample preparations, the enamel surface was polished and blocks were cross-sectioned at one millimeter from the edge of each tooth sample to obtain 57 blocks of 3×4×3 millimeters from the buccal (N = 10), occlusal (N = 12), lingual (N = 5), mesial (N = 19), distal (N = 15) surfaces. In addition, 15 blocks from root surfaces were obtained to be used as controls. Blocks were submitted to baseline microhardness analysis using a microhardness tester (IndentaMet 1100 Series, Buehler Ltd., Lake Bluff, IL, USA) with a knoop diamond under a load of 25 grams for 10 seconds. Three indentations spaced 100 µm away from each other were made. Artificial caries lesions were created by immersing each enamel block in 24 mL of demineralizing solution (1.3 mmol/L Ca, 0.78 mmol/L P, 0.05 mol/L acetate buffer, 0.03 µgF/mL, pH 5.0) at 37°C for 16 hours [20]. This method produces a subsurface enamel demineralization without surface erosion [21]. Surface microhardness was measured again by another three indentations created right underneath the initial ones. Carious lesions were exposed to a fluoride solution made from toothpaste containing sodium fluoride (0.15% w/v fluoride ion; Aquafresh Extreme Clean, GlaxoSmithKline, Brentford, Middlesex, UK) to bring microhardness values back to baseline levels. Surface microhardness was measured one more time and other three indentations created right underneath the previous ones were obtained.

After remineralization of the enamel block surfaces, a pH-cycling protocol was implemented to test the dinamic effect of fluoride on a high caries environmental challenge [11]. The cycle alternated between a demineralizing solution (2.0 mmol/L Ca and P, 0.075 mol/L acetate buffer, 0.03 µgF/mL. pH 4.7; 0.75 mL/mm^2^) and a remineralizing solution (1.5 mmol/L Ca, 0.9 mm0l/L P, 0.15 mol/L KCl, 0.02 mol/L cacodylate buffer, 0.04 µg F/mL, pH 7.0; 0.25 mL/mm^2^) during 14 days. At 8 AM, all specimens were immersed in the remineralizing solution; at 12 PM, specimens were washed with deionized water and immersed in the demineralizing solution; at 2 PM, specimens were washed and immersed in the same remineralizing solution used at 8 AM; at 4 PM, specimens were washed and immersed in a new remineralizing solution, in which they were kept until 8AM the next day, when the remineralizing solution was replaced again as a new cycle started. Surface microhardness was measured one last time and the measurement for the other three indentations created right underneath the ones previously obtained.

The baseline microhardness and rates of change of microhardness scores after artificial caries creation, fluoride application, and pH-cycling were calculated. Each measurement had three replicates and the mean of the three values was calculated and used in all analyses. Phenotypes were defined based on dicothomous groups (baseline values or rate changes above or below the average of the group). These phenotypes were tested for association with the genetic variants in the enamel formation genes. Six SNPs previously studied [2]–[4] were added in the analysis including rs17878486 in *AMELX*, rs3790506 and rs2337360 in *TUFT1*, rs34538475 in *AMBN*, rs3796704 in *ENAM*, and rs134136 in *TFIP11*. The differences in genotype and allele frequencies between high and low rate of change for microhardness were tested by Fisher’s exact test. We performed correlation tests to help interpret the changes in enamel microhardness observed.
